# Response: Commentary: Incubation and Intuition in Creative Problem Solving

**DOI:** 10.3389/fpsyg.2017.00465

**Published:** 2017-03-30

**Authors:** Kenneth J. Gilhooly

**Affiliations:** ^1^Psychology Department, University of HertfordshireHatfield, UK; ^2^Department of Clinical Sciences, Brunel University LondonLondon, UK

**Keywords:** creative thinking, problem solving, incubation effect, intuition, cognitive theory

Yuan and Shen (2016), in a commentary on Gilhooly's (2016) explanation of incubation in terms of Unconscious Work, suggested that a Dual Process account may be a useful alternative explanation. Although Dual Process theories have been useful in studies of decision making (Greene, 2007) and reasoning (Evans, 2008), I will argue that the usefulness of this approach to explaining incubation is not clear.

Dual Process theories vary in their details (Smith and DeCoster, 2000; Evans, 2008; Stanovich, 2009) but arguably, there is a core “modal model” of Dual Process thinking, broadly as put forward by Kahneman (2011), according to which System 1 processes are parallel, implicit, and unconscious, and do not involve working memory or executive control and System 2 processes are sequential, explicit, conscious, under executive control, and do load working memory.

In experimental studies, incubation periods, by definition, are periods in which a target task is to be set aside and not consciously processed. During this period only unconscious processes (System 1) should be operative on the target task and it is hard to see, on the generally accepted definitions of incubation and Dual Process Theory, how the System 2 component of Dual Process Theory could be directly involved during the incubation period unless Intermittent (conscious) Work on the target task is accepted as a possible explanation. On the basis of a number of studies looking at this possibility, I feel we have been able to rule out the Intermittent (conscious) Work hypothesis (Gilhooly et al., 2012, 2013).

The idea of Conscious Work and Unconscious Work occurring at different stages of creative thinking, as classically proposed by Wallas (1926) and Poincare (1910), is consistent with a Dual Process Theory, in that System 2 is evident in the initial conscious phase (Wallas's Preparation Stage) and the final Verification Stage, while System 1 is involved in the unconscious Incubation stage. If this is what Yuan and Shen were proposing I would agree that there is a reasonable mapping of the relatively new System 1 and 2 terminology onto the classical Stages terminology, and such a mapping is of value in that it enables linkages to be made between creative problem solving and other areas of thinking research, such as reasoning and decision making.

The nature and the duration of initial conscious (System 2) work in the Preparation stage have been widely acknowledged to influence subsequent unconscious (System 1) processing during the Incubation stage e.g., the classic analyses of Wallas and Poincare proposed that delayed Incubation required extensive preliminary work to be effective. Similarly, the nature of the conscious work on the interpolated task during the Incubation period is recognized to be a factor in determining the effectiveness of unconscious work on the target task during the incubation period (Gilhooly et al., 2013), in that both may be seen as competing for limited cognitive resources. So, in the standard approach, System 2 processes can affect System 1 processes. In the other direction, System 1 processes are important in determining how the target task is initially represented and so influence subsequent System 2 processes.

However, Yuan and Shen seem to be proposing parallel System 1 and 2 processes, both bearing on the target task at the same time (rather than System 2 dealing with an interpolated task and System 1 dealing simultaneously with the target task during the incubation period). They propose that with a very light loading interpolated task e.g., rest, incubation becomes “…an entirely conscious process.” This implies that the target task is being processed consciously during such an incubation period, which contradicts the definition of incubation as a period in which the target task is not to be consciously addressed, and seems to be re-stating the Intermittent Work hypothesis in a more extreme form, as a Continuous Work Hypothesis, at least for the case where no explicit interpolated task is given during the incubation period. Indeed, they also state that “…the conscious process underlying incubation is not intermittent and always goes with unconscious processes during the whole incubation period.” It is not clear if Yuan and Shen mean that the continuous conscious processes are addressing the target task or the interpolated task or both. If the conscious process is only addressing the target task and is continuous, then there is no incubation (and how could interpolated tasks be executed without conscious attention?); if the conscious processes are addressing solely the interpolated task (as instructed), then their effects on the unconscious processes occurring at the same time are incidental (as would be widely accepted). A third possibility is of intermittent conscious work on the target task, which Yuan and Shen seem to rule out (and is one which our data do not support, Gilhooly et al., 2012, 2013).

We have not addressed the issue of incubation periods with no specified interpolated tasks or with only very light interpolated tasks in our experimental studies and it may be that such conditions do promote mind wandering. Mind wandering could lead to a return of the target task goal to consciousness and some subsequent intermittent conscious processing. However, Baird et al. (2012) reported that task relevant thoughts during mind wandering in an incubation period did not influence post incubation performance on Uses tasks, but greater mind wandering was nevertheless beneficial. Mind wandering could be viewed as an unconsciously driven associative process (with conscious reportable outputs) which re-distributes activation over elements in long term memory, which in turn makes novel links more available when the task is resumed after the incubation period. Mind wandering thus could be seen as a type of System1 activity deliberately engaged in during incubation periods with beneficial results. If mind wandering is the kind of conscious process, that Yuan and Shen are proposing, as always underlying incubation, I can agree that this is a viable hypothesis. However, Yuan and Shen do not explicitly mention mind wandering or similar terms, such as daydreaming, so it is not clear whether my interpretation of their outline model matches what the authors intend.

## Author contributions

KG confirms being the sole contributor of this work and approved it for publication.

## Funding

This paper is based on research funded by grants from UK Economic and Social Research Council (RES-000-22-2191) and Leverhulme Trust (F008281G) to KG.

### Conflict of interest statement

The author declares that the research was conducted in the absence of any commercial or financial relationships that could be construed as a potential conflict of interest.
